# Reductive stress in mitochondria isolated from the carotid body of type 1 diabetic male Wistar rats

**DOI:** 10.14814/phy2.70016

**Published:** 2024-09-18

**Authors:** Hector R. Tejeda‐Chavez, Sergio Montero, Alfredo Saavedra‐Molina, Monica Lemus, Julio B. Tejeda‐Luna, Elena Roces de Alvarez‐Buylla

**Affiliations:** ^1^ Faculty of Medicine Colima of University Colima Mexico; ^2^ Department of Neuroendocrinology, University Center of Biomedical Research Colima University Colima Mexico; ^3^ Institute of Biological Chemistry Research UMSNH Morelia Michoacán Mexico

**Keywords:** carotid body, glutathione, mitochondria, reductive stress, streptozotocin‐induced diabetes

## Abstract

The carotid body (CB) senses changes in arterial O_2_ partial pressure (*p*O_2_) and glucose levels; therefore, it is key for the detection of hypoxia and hypoglycemia. The CB has been suggested to detect *p*O_2_ through an increase in reactive oxygen species (ROS) in the mitochondria. However, the mechanism protecting the chemoreceptor cells and their mitochondria from ROS and hyperglycemia is poorly understood. Here we measured glutathione levels in CB mitochondria of control and in streptozotocin (STZ)‐induced type 1 diabetic male Wistar rats. We found a dramatic reduction in total glutathione from 11.45 ± 1.30 μmol/mg protein in control rats to 1.45 ± 0.31 μmol/mg protein in diabetic rats. However, the ratio of reduced to oxidized glutathione, a measure of the redox index, was increased in diabetic rats compared to controls. We conclude that the mitochondria of CB chemoreceptor cells in type 1 diabetic male Wistar rats were likely under glutathione‐reducing stress.

## Graphical abstract

Glutathione‐reducing stress in mitochondria isolated from the carotid body of type 1 diabetic male Wistar rats.

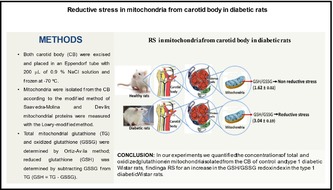

## 1 INTRODUCTION

Diabetes mellitus (DM) is an endocrine disease, associated with multiple metabolic disorders, all sharing the common phenotype of hyperglycemia. According to pathophysiology, distinct types of DM are caused by a complex interaction of genetic and environmental factors (Powers et al., 2022, Skyler et al., 2017).

The carotid body (CB) plays a key role informing the brain of the levels of partial pressure of oxygen (*p*O_2_), partial pressure of carbon dioxide (*p*CO_2_), hydrogen potential (pH), osmolarity, temperature (Eyzaguirre & Zapata, 1984; Kumar & Prabhakar, 2012), and glucose (Alvarez‐Buylla & de Alvarez‐Buylla, 1988; Koyama et al., 2000; Kumar & Prabhakar, 2012; Pardal & Lopez‐Barneo, 2002) in arterial blood entering the cephalic circulation. The CB is extensively vascularized (McDonald & Blewett, 1981) and innervated by the Hering's nerve (De Castro, 1926). The CB is strategically located in the initiation of the cephalic circulation, at the initial segment of the internal carotid artery, just past its bifurcation from the common carotid artery (Alvarez‐Buylla & de Alvarez‐Buylla, 1988; Kumar & Prabhakar, 2012). The CB is composed of clusters of sustentacular glia‐like cells, and glomus or chemoreceptor cells (Joyner et al., 2018; Lopez‐Barneo et al., 2009).

An anoxic stimulus—by the transient local application of sodium cyanide (NaCN) to vascularly isolated carotid sinus (CS)—induces rapid hyperventilation and hyperglycemia. NaCN in the CB also increases glucose uptake by the brain and glucose output by the liver (Alvarez‐Buylla & de Alvarez‐Buylla, 1988; Alvarez‐Buylla & Roces de Alvarez‐Buylla, 1994). Interestingly, in streptozotocin (STZ)‐induced type 1 diabetic rats, which had almost three times the normal levels of circulating glucose and the stimulation with NaCN in the isolated CS also induced a similar increase in circulating glucose (Tejeda‐Chavez et al., 2010).

In other studies, the glucose infusion required to maintain hypoglycemia or euglycemia in dogs was significantly increased in the absence of the CB, further showing a link between CB function and glucose regulation (Koyama et al., 2000). Furthermore, it has been shown that low‐glucose levels in the CB, is associated with the inhibition of a K^+^ channels and membrane depolarization, the influx of Ca^2+^ through voltage‐gated Ca^2+^ channels, and the release of dopamine (Pardal & Lopez‐Barneo, 2002). It has been suggested that CB glucose sensing could be altered in diabetic patients, particularly those under insulin treatment (Gao et al., 2014).

Glutathione (GSH) (composed by γ‐L‐glutamyl‐L‐cysteinyl‐glycine) is a tripeptide, which has multiple biological roles including protection against reactive oxygen (ROS) and reactive nitrogen species (RNS) (Lushchak, 2012). GSH is an electron donor that becomes oxidized to form glutathione disulfide (GSSG) through catalysis of the GSH peroxidase (GPX) enzyme, and as a direct reaction to ROS (Diaz Vivancos et al., 2010; Forman et al., 2009; Giustarini et al., 2016; Lushchak, 2012; Wall et al., 2014). GSSG is reduced to GSH through the glutathione reductase enzyme (GRE). Glutathione, in its reduced form (GSH), increases the synthesis of NO due to an increase in nitric oxide synthase (NOS) activity, possibly to protect the oxidative effect of ROS and RNS. NO also increases GSH synthesis and redox, through the transcription of γ‐glutamylcysteine synthetase, glutathione reductase and glutathione synthetase (Aquilano et al., 2014; Zhang et al., 2019). Glutathione, therefore, regulates and maintains the thiol‐redox status in cells (Giustarini et al., 2016). The GSH to GSSG molar ratio is considered a powerful index of oxidative stress and disease risk (Giustarini et al., 2016). GSH is present in multiple cellular compartments including the nucleus, the cytoplasm and the mitochondria (Diaz Vivancos et al., 2010).

Reductive stress (RS), is also characterized by an elevation of nicotinamide adenine dinucleotide phosphate (NAD(P)H) and/or glutathione or GSH/GSSG ratio (Wendel, 1987); and a low NAD+/NADH ratio that can result in pathologies such as diabetes (Chiao et al., 2021); RS can impair mitochondrial function by decreasing maximal mitochondrial respiration and increasing hydrogen peroxide (H_2_O_2_) production (Peris et al., 2019; Singh et al., 2015). It has been suggested that the antioxidative properties of RS can become disease‐inducing (Ma et al., 2020). It has been shown that GSH plays a key role in the redox environment of chemosensory cells in the CB (Garcia‐Ruiz et al., 1995; Sanz‐Alfayate et al., 2001). Conversely, a high NAD+/NADH ratio or decreased of GSH/GSSG, an increase of ROS and/or depletion of the enzymatic and non‐enzymatic antioxidant system is characterized by oxidative stress (Chiao et al., 2021).

In addition, a significant reduction in GSH concentration, due to low GRE activity, has been observed in kidney cell mitochondria from aged STZ‐induced diabetic rats, and this could be associated with “accelerate aging” (Ghafourifar & Saavedra‐Molina, 2005, Perez‐Gallardo et al., 2014). Interestingly, in red blood cells from type 1 diabetic patients, fasting plasma glucose, and GSH levels become de‐correlated compared with control subjects (Likidlilid et al., 2007).

Here, we studied the effects of short‐term hyperglycemia on the levels of TG, GSH, and GSSG in mitochondria isolated from the CB of adult rats. Short‐term hyperglycemia was induced with streptozotocin (STZ) to type 1 diabetes.

## 2 MATERIALS AND METHODS

### 2.1 Ethics

All experiments were performed in accordance with the National Institutes of Health (NIH) Guidelines for the Care and Use of Laboratory Animals, and the Bioethics as Biosecurity Committee of the School of Medicine and University Center for Biomedical Research (CUIB) of the University of Colima, Mexico (no. 2020–06), approved in February of 2020.

### 2.2 GSH kits and anesthetic

GSH + GSSG/GSH Assay Kit (Colorimetric) (ab239709, Sigma, Mex) (Ortiz‐Avila et al., 2015). Sodium Pentobarbital (Q‐7972‐004, Sedalpharma, Pet's Pharma, Mex) (3 mg/100 g/0.3 mL in 0.9% NaCl) (Alvarez‐Buylla & de Alvarez‐Buylla, 1988).

### 2.3 Animals and experimental protocol

For all experiments we used 16‐week‐old male Wistar rats (they are more susceptible to diabetes than females) (Poret et al., 2021) weighing 250–300 g, kept in polyethylene cages, at a temperature 22–24°C, with a 12‐h light/dark cycle, fed a standard rodent diet and given water ad libitum. The 20 rats were randomly divided into 10 control and 10 experimental streptozotocin (STZ) treated (type 1 diabetic) male rats. All rats were fasted (food removed, but free access to water) for 12 h beginning in the evening (8 pm) the day before the experiments. The following morning (8 am) a drop of blood was drawn from the tail vein to make sure the blood glucose levels were within normal values (< 100 mg/dL). Rats in the experimental group were injected with a single dose of SZT (S0130, Sigma‐Aldrich, Toluca, Mex., 60 mg/kg [IP] in 1 mL of 0.9% NaCl solution). The control group was injected with the same volume [IP] of 0.9% NaCl solution. The following night at 8 pm all rats were again fasted for 12 h and glucose was measured in the tail vein the following morning (8 am). This procedure with a morning glucose measurement was repeated for 3 days. All SZT‐treated rats developed hyperglycemia above 300 mg/dL by day three, levels consistent with a diabetic state (Moree et al., 2013; Szkudelski, 2001). The average glucose concentration in the control rats was 84.5 ± (SD) 2.5 mg/dL, whereas in the STZ‐treated rats was 321 ± (SD) 3 mg/dL. Glucose concentration was determined with a Glucometer (Accu Chek Performa, Roche Lab, Germany). We observed body weight loss in all diabetic rats.

### 2.4 Extirpation of the carotid body

**FIGURE 1:**
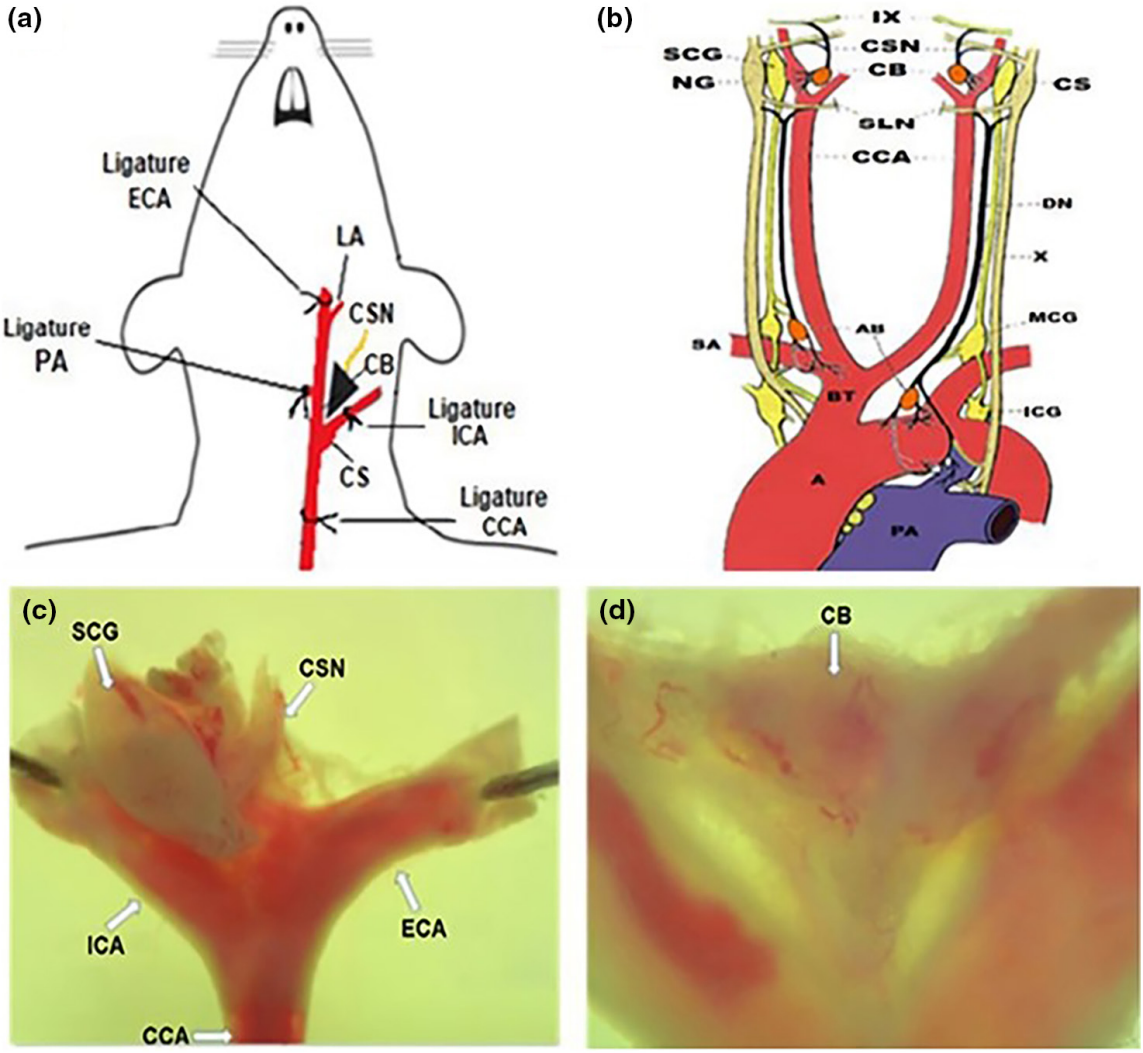
(a) Schematic of carotid body excision. Anatomical location of the CB in the internal carotid arteries above the bifurcation of the common carotid arteries. The CB was surgically isolated in anesthetized rats before its removal. Anatomy of the common carotid artery and its bifurcations (5). (b) Large arterial vessels: Aorta (with oxygenated blood to irrigate the brain, the rest of the body), and pulmonary artery (with deoxygenated blood, to oxygenate the lungs). The aortic and carotid bodies are illustrated (Modified from the original drawing donated by Nonidez JF, unpublished). (c) Photomicrography with the dissection of the CB, and the superior cervical ganglion to be extirpated, ECA and ICA fixed by entomological pins in a Petri dish with an agar gum film as base in saline solution. (d) A higher resolution of the CB is observed, suspended in the bifurcation of the common carotid artery at the origin of the internal carotid artery, showing its blood supply. A, aorta; AB, aortic body; BT, brachiocephalic trunk; CB, carotid body; CCA, common carotid artery; CS, carotid sinus; CS, carotid sinus; CSN, carotid sinus nerve; DN, depressor nerve; ECA, external carotid artery; ICA, internal carotid artery; ICG, inferior cervical ganglion; LA, lingual artery; MCG, middle cervical ganglion; NG, nodose ganglion; PA, pulmonary artery; PhA, pharyngeal artery; SA, subclavian artery; SCG, superior cervical ganglion; SLN, superior lingual nerve; IX, branch of the glossopharyngeal nerve; X, branch of the vagus nerve or cranial nerve. Permanent carotid ligature is indicated.

In order to cleanly isolate the CB, rats were anesthetized with sodium pentobarbital (3 mg/100 g IP), and kept on endotracheal respiration to avoid ischemia. Consistent stable anesthesia was maintained throughout the extirpation procedure (~30 min) by an IP infusion (2 drops / min sodium pentobarbital at a concentration of 1.8 mg/100 mL in 0.9% NaCl) (Alvarez‐Buylla & Roces de Alvarez‐Buylla, 1994); Anesthesia was monitored by means of palpebral and leg prick reflexes. Buprenorphine (0.03 mg/kg sc) was used as an analgesic. Rat body temperature was maintained at 37°C with a hot pad. Rats were placed on a surgical table in a dorsal decubitus position. A 2 mm midline incision from the ventral side of the neck to the anterior sternum was used to expose the trachea. A respiratory cannula (I.D. 2 mm, E.D. 3 mm) (Guarner & Alvarez‐Buylla, 1991) connected to an artificial ventilator (Ugo Basile, Stoelting, Wood Dale, IL, USA) was introduced into the trachea. Arterial gases (*p*O_2_, *p*CO_2_), and pH levels were determined by a gas analyzer (Micro 13, Instrumentation Laboratory, Lexington, MA) in mmHg and absolute units, respectively. Blunt dissections of the sternohyoid, omohyoid, and sternomastoid muscles were made to expose 2.5 cm of the common carotid artery and the carotid bifurcation. The lingual and the common, external, and internal carotid arteries were dissected with glass hooks. The common and external carotid arteries (1.5 cm before the bifurcation) were ligated bilaterally from both sides, to stop the circulation (Figure 1a,b). Both bifurcations (surgical specimens) containing the CB and upper cervical ganglion were excised and the surgical specimens were placed in 0.9% NaCl solution in a Petri dish coated with Silgar resin (Silicone RTV‐2, Rhôme Poulenc) (Figure 1c) (Hernandez‐Leal et al., 2018).

After removing the cervical superior ganglion, the surgical specimen was pinned to the Silgar‐coated dish (under the microscope Figure 1d). Both, right and left CBs were dissected, extracted and placed in an Eppendorf tube with 200 μL of 0.9% NaCl solution and immediately frozen at −70°C. At the end, the rats were euthanized by decapitation (Waynfort & Flecknell, 1995) under anesthesia.

### 2.5 Mitochondria isolation

Mitochondria were isolated from the CB according to the modified method of Saavedra‐Molina and Devlin, 1997. The isolated CBs from control and diabetic rats were thawed and placed in 2 separate tubes in buffer A (210 mM mannitol, 70 mM sucrose, 1 mM EGTA, 0.5% albumin, 10 mM 3‐(N‐morpholino) propanesulfonic acid (MOPS), at pH 7.4) at 4°C (Ortiz‐Avila et al., 2015). The 20 CBs from the 10 rats in each group were manually homogenized in two separate tubes (for separate measurements) with a glass rod homogenizer (Thomas Scientific Glass Homogenizer and Teflon Pistil, Swedesboro, NJ, USA) and centrifuged at 4000 ×*g* for 10 min at 4°C. The supernatant was separated and centrifuged at 9000 ×*g* for 10 min at 4°C. The pellet was resuspended in buffer B (210 mM mannitol, 70 mM sucrose, 0.5 mM EGTA, 10 mM K2HPO4, 10 mM MOPS at pH 7.4) at 4°C (Ortiz‐Avila et al., 2015) and centrifuged at 10,000 ×*g* for 10 min at 4°C (Sims, 1990). The mitochondrial pellet (2–3 mm diameter) was isolated from the Eppendorf tube for subsequent analysis. Mitochondrial protein was measured with the Lowry‐modified method and calibrated with bovine serum albumin (Lowry et al., 1951).

### 2.6 Glutathione determination

Total mitochondrial glutathione (TG) was determined as previously described (Ortiz‐Avila et al., 2015). Isolated mitochondria were treated with 5% v/v sulfosalicylic acid and centrifuged at 7800 ×*g* for 10 min. TG (GSH + GSSG) were measured in a cuvette containing 90 μL of the supernatant in 0.1 M Na^+^ phosphate buffer at pH 7.5, 3 mM of 5,5′‐dithiobis (2‐nitrobenzoic acid) (DTNB), and 0.115 units/mL of glutathione reductase in a final volume of 1 mL. After 5 min of incubation at room temperature, 2 nM NADPH was added. The absorbance at 412 nm was converted to a GSH concentration, using a standard curve with a known amount of GSH (Akerboom & Sies, 1981). To determine the levels of oxidized glutathione (GSSG), GSH was removed by a 1 h incubation with 3% v/v 4‐vinyl pyridine at room temperature.

### 2.7 Statistical analysis

Results are expressed as mean ± SD. The experiments were repeated four times (biological replicates) by duplicate (technical replicates). The student's *t*‐test for two independent samples was used to compare the four experiments (20 CBs) in each group of control and diabetic rats. It is important to note that for the four experiments we pooled the 20 CBs from 10 rats, in each group (control and diabetic rats). According to our results, we considered statistically significant differences when the confidence level was above 95% and a *p*‐value <0.01.

## 3 RESULTS

**FIGURE 2:**
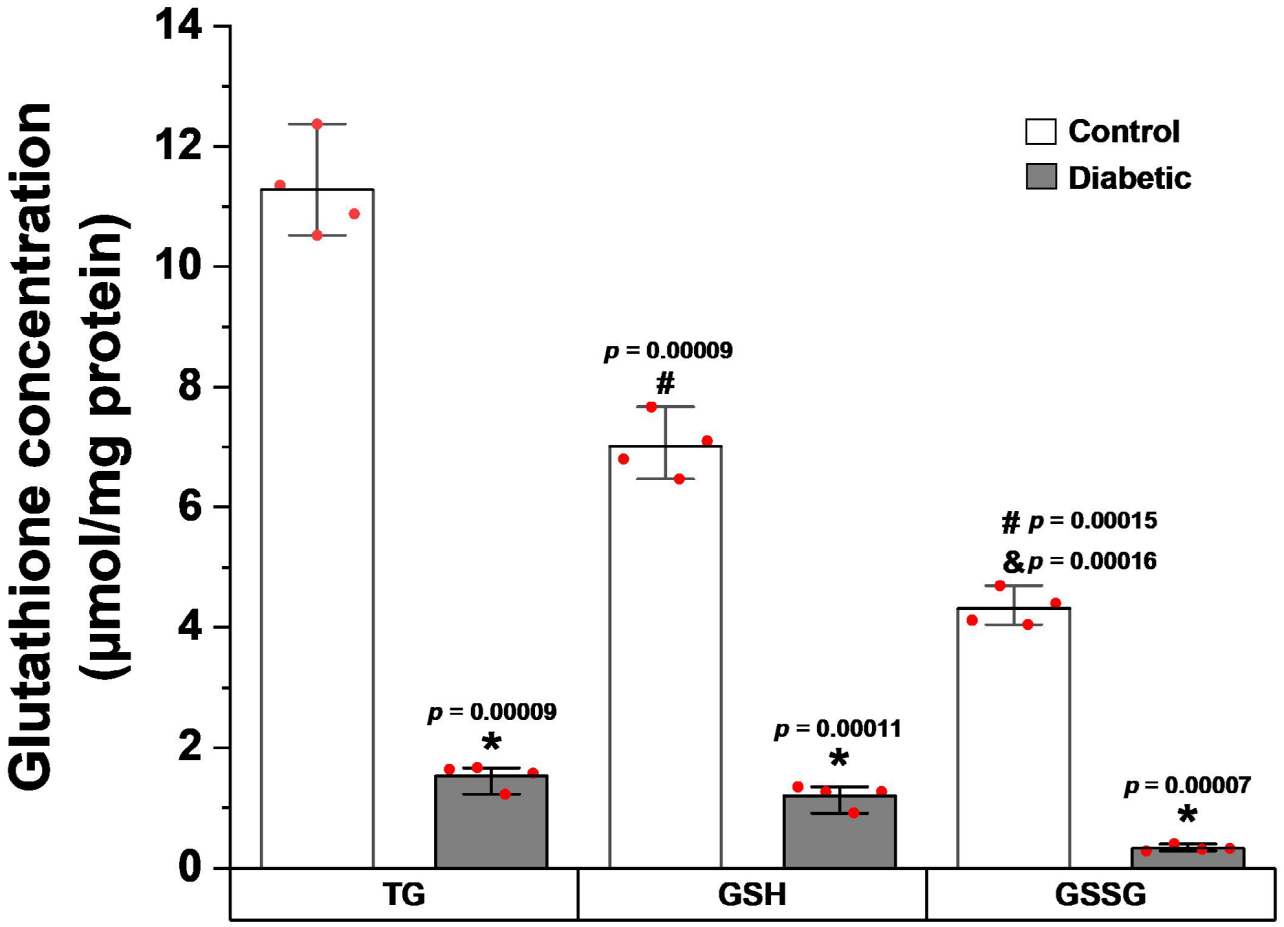
Concentration of total glutathione (TG), reduced glutathione (GSH), and oxidized glutathione (GSSG) in the mitochondria of the CB in control and type 1 diabetic Wistar rats. Values expressed as means ± standard deviation. *Statistically significant in both groups of rats (*n*=4/group of 20 CB), *p* < 0.01 versus control rats values; #*p* < 0.01 versus TG values; and *p* < 0.01 versus GSH values. Student's *t*‐ test (*t* test) for two independent samples.

TG and oxidized glutathione (GSSG) in mitochondria from the CBs were measured in the control and in STZ hyperglycemic rats as a model of Type 1 diabetes. Reduced glutathione (GSH) was determined by subtracting GSSG from TG (GSH = TG–GSSG). TG in the CB from mitochondria of control rats was 11.28 ± 0.80 μmol/mg protein. TG in the CB from mitochondria of diabetic rats was dramatically low to 1.53 ± 0.20 μmol/mg protein; it was significant (*p* < 0.00009, Figure 2). Similarly, GSH and GSSG were significantly low in diabetic rats compared to controls: GSH was 7.01 ± 0.51 in controls vs 1.20 ± 0.19 μmol/mg protein in diabetic rats; it was significant (*p* < 0.00011, Figure 2) and GSSG was 4.32 ± 0.29 in controls vs 0.33 ± 0.05 μmol/mg protein in diabetic rats; it was significant (*p* < 0.00007, Figure 2).

**FIGURE 3:**
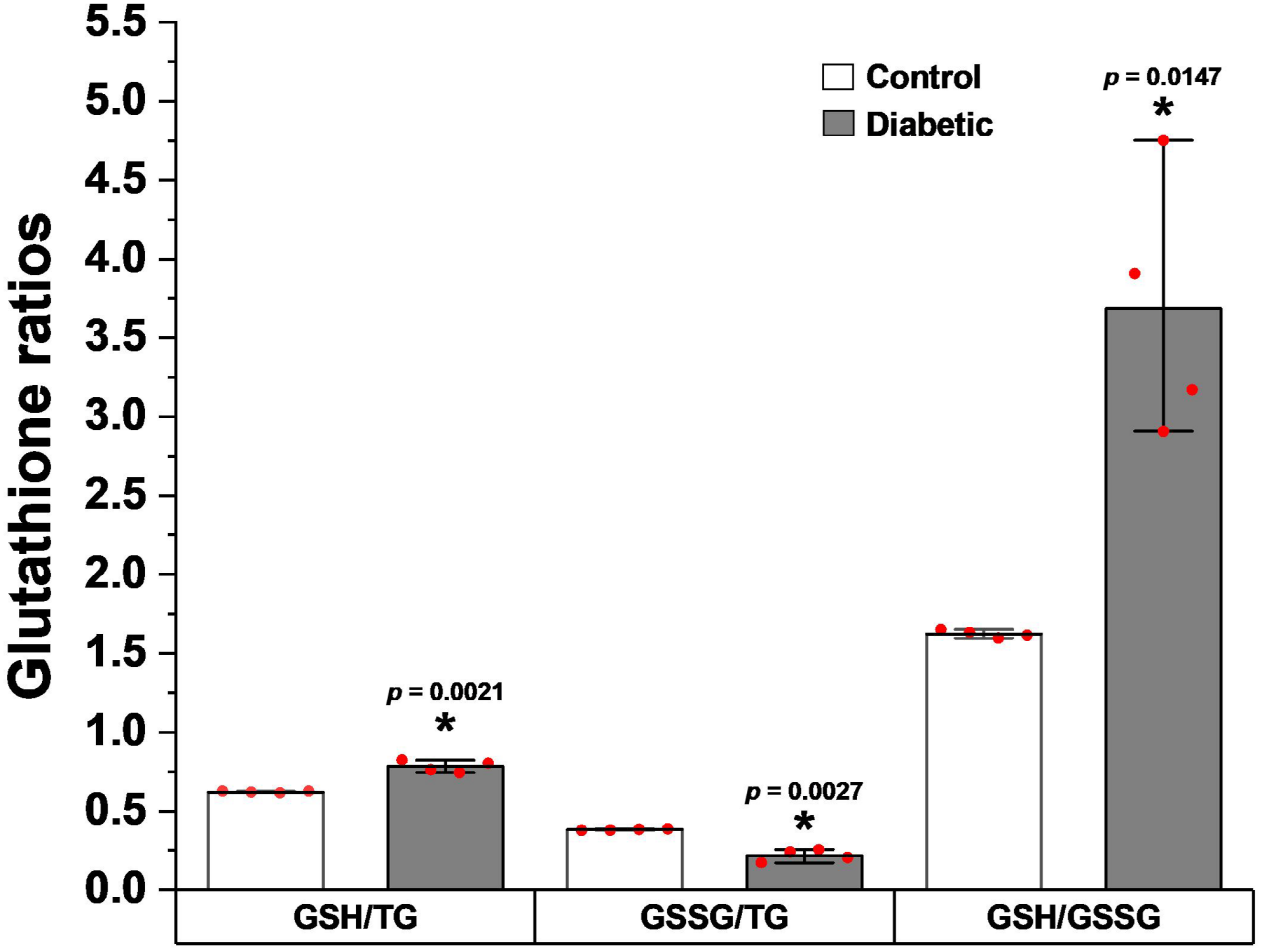
GSH/TG, GSSG/TG, and GSH/GSSG Indices in the mitochondria of the carotid body in control and type 1 diabetic Wistar rats. Values expressed as means ± standard deviation. *Statistically significant in the same groups of rats, *p* < 0.01 versus control rat values. GSH, reduced glutathione; GSSG, oxidized glutathione; TG, total glutathione. Student's *t*‐test (*t* test) for two independent samples.

The ratio of GSH over TG (GSH/TG), is an indicator of the balance of the reduction state of the glutathione system in the mitochondria, was increased from 0.62 ± 0.00 in control to 0.78 ± 0.04 in diabetic rats; it was significant (*p* < 0.00211, Figure 3). In contrast, the ratio of GSSG over TG (GSSG/TG), is an indicator of the balance of the oxidized state, was decreased in control rats (0.38 ± 0.00) compared to diabetic (0.22 ± 0.04), it was significant (*p* < 0.00273, Figure 3). The ratio of reduced (GSH) over oxidized (GSSG) (GSH/GSSG) is frequently used as an indicator of the balance of overall redox state. In control rats, the GSH/GSSG ratio was 1.62 ± 0.01, but was almost twice as high in the diabetic rats (3.68 ± 0.83), it was significant (*p* < 0.01472, Figure 3).

## 4 DISCUSSION

Using STZ to model type 1 diabetes in rats we show that hyperglycemia resulted in changes in the redox index in the mitochondria isolated from the CBs. We found much lower TG, GSH, and GSSG concentrations in the mitochondria of CB of diabetic rats compared with controls rats. Changes in the ratio of TG, GSH, and GSSG indicated an increase in mitochondrial redox index in the diabetic rats compared to controls. Our results suggest that the antioxidant mechanism in the mitochondria of the CB in type 1 diabetic rats is likely in RS compared to controls. This was manifested by an increase in GSH/TG and in the GSH/GSSG redox indices, and a significant reduction in the GSSG/TG index. However, these indices are an indicator of reductive or oxidative stress, we consider it pertinent to measure other indicators (discussed below) of reducing stress, to complement our results, which will strengthen our hypothesis, that the mitochondria of the CB of (STZ)‐induced type 1 diabetic rats are under glutathione‐reducing stress as a compensatory mechanism to hyperglycemia, which could damage CBs chemoreceptors and alter their physiology mechanisms.

Cellular and mitochondrial redox homeostasis is connected and regulated by redox‐sensitive couples: glutathione (GSH)/glutathione disulfide (GSSG), NADP+/NADPH, and NAD+/NADH, as well as by ROS. NADH, and NADPH can be produced in both cytosol and mitochondria. Because the reactions involved with the redox pairs are interconnected, disturbances of the redox couples in a specific subcellular compartment will affect the whole cell redox homeostasis and ROS generation/scavenging (Yu et al., 2014). Given the importance of glutathione as one of the main antioxidant systems (Sanchez‐Duarte et al., 2021), the increased GSH/GSSG index in the mitochondria of the CB of type 1 diabetic rats is likely due to the short‐term hyperglycemia. We hypothesized that short‐term hyperglycemia in the CB of type 1 diabetic rats saturates the glycolysis and pentose phosphate metabolic pathways, which increases NADPH generation (unfortunately, we could not determine levels of NADPH due to the small size of the CB). The relatively high levels of GSH with respect to TG observed is likely a compensatory response to protect the cell from hyperglycemic damage. However, if this compensatory response reaches a maximum and the redox buffer capacity is exceeded, RS occurs to decrease ROS (Xiao & Loscalzo, 2020). Luc et al., (2019), observed that hyperglycemia upregulate chronic inflammation markers and contributes to increased ROS generation, causing vascular dysfunction. Such perturbations in the redox state could be associated with the re‐establishment of homeostatic levels toward RS (Ma et al., 2020). Chronic hyperglycemia causes oxidative damage in the skeletal muscle of STZ‐induced diabetic rats. In this same study, administration of Nicorandil increases glutathione levels, resulting in decreased oxidative damage by decreasing lipid peroxidation and an increase in insulin tolerance (Sanchez‐Duarte et al., 2021).

The mitochondria are the primary intracellular site for oxygen (O_2_) consumption and the largest source of ROS (Mari et al., 2009). Gores et al. (1989), induced anoxia in rats with potassium cyanide (KCN) and iodoacetic acid to block mitochondrial respiration and ATP production (chemical hypoxia) resulting in RS, the formation of toxic oxygen species, and lethal cell injury. Moreover, in the lungs of a diabetic rat model, in which the polyol pathway is activated, the respiratory complex I‐IV, and ROS in mitochondria are increased (Wu et al., 2017). These observations support that in multiple tissues, RS occurs during hyperglycemia. Electrons from aerobic glycolysis are stored in NADH for O_2_ oxidation and ATP generation. An aberrant increase in the GSH/GSSG ratio due to hyperglycemia leads to RS (Yu et al., 2014) that could trigger mitochondrial dysfunction and cytotoxicity in cardiac muscle (Ma et al., 2020; Zhang et al., 2012). Our quantifications of TG and GSSG in the hyperglycemic (type 1 diabetic) rats revealed a significant increase in GSH/GSSG ratio compared to control rats. This observation suggests that after a short period (3 days) of hyperglycemia, the mitochondria of this important chemoreceptive organ are under RS. Our observation that the RS is increased in the isolated mitochondria from the CB could result in dysfunction of these key chemoreceptors and aberrant signaling to the brain on the composition of the blood (including glucose levels in the diabetic rat) entering the cephalic circulation.

### 4.1 Limitations

The levels of NADP+/NADPH and NAD+/NADH, as well as by ROS in mitochondria isolated from the CBs of healthy and diabetic rats were not determinated.

Further investigation of GSH/GSSG indices, and NAD+/NADH, as well as by ROS in the mitochondria of the CBs of obese rats represents an important future direction, as it will allow us to analyze the effects on glucose metabolism and to understand the pathophysiological mechanism of mellitus diabetes.

## 5 CONCLUSION

In our experiments, we quantified the concentrations of total and oxidized glutathione in mitochondria isolated from the CB of control and type 1 diabetic male Wistar rats. We found that the concentration of GT was tenfold lower in the type 1 diabetic male Wistar rats. Furthermore, in this same experimental group we showed interestingly an altered redox state with a clear tendency toward reduced status, due to a significant increase in the GSH/GSSG and GSH/GT redox indices and significant decrease in the GSSG/TG index; which infer us a probable glutathione‐reducing stress, as a compensatory mechanism to hyperglycemia.

## AUTHOR CONTRIBUTIONS

H.R.T.‐C., J.B.T.‐L., and E.R.AB.: Conceived and designed research; H.R.T.‐C. and S.M.: Performed experiments to remove the CBs; H.R.T.‐C. and A.SM.: Performed experiment to isolating mitochondria from the CBs; H.R.T.‐C., S.M., M.L., and J.B.T.‐L.: Analyzed data; H.R.T.‐C., S.M., J.B.T.‐L., and E.R.AB.: Interpreted results of experiment; S.M. and M.L.: Prepared figures; H.R.T.‐C., S.M., and J.B.T.‐L.: Drafted manuscript; H.R.T.‐C., S.M., J.B.T.‐L., and E.R.AB.: Edited and revised manuscript; H.R.T.‐C., S.M., A.S.M., M.L., J.B.T.‐L., and E.R.AB.: Approved final version of manuscript.

## FUNDING INFORMATION

This work was supported by research (no. DSA/103.5/15/10869) from Program for Teaching Professional Development (PRODEP) from Secretary of Public Education.

## CONLICT OF INTEREST STATEMENT

The authors declare no conflicts of interests, financial or otherwise, to disclose.

## ETHICS STATEMENT

The study protocol was approved by the Bioethics as Biosecurity Committee of the School of Medicine and University Center for Biomedical Research (CUIB) of the Universityof Colima, Mexico (No. 2020‐06), approved in February of 2020.

## Data Availability

All data generated and analyzed during the current study are available from the corresponding author upon reasonable request.

## References

[phy270016-bib-0001] Akerboom, T. P. , & Sies, H. (1981). Assay of glutathione, glutathione disulfide, and glutathione mixed disulfides in biological samples. Methods in Enzymology, 77, 373–382. 10.1016/s0076-6879(81)77050-2 7329314

[phy270016-bib-0002] Alvarez‐Buylla, R. , & de Alvarez‐Buylla, E. R. (1988). Carotid sinus receptors participate in glucose homeostasis. Respiration Physiology, 72, 347–359. 10.1016/0034-5687(88)90093-x 3406554

[phy270016-bib-0003] Alvarez‐Buylla, R. , & Roces de Alvarez‐Buylla, E. (1994). Changes in blood glucose concentration in the carotid body‐sinus modify brain glucose retention. Brain Research, 654, 167–170. 10.1016/0006-8993(94)91585-7 7982092

[phy270016-bib-0004] Aquilano, K. , Baldelli, S. , & Ciriolo, M. R. (2014). Glutathione: New roles in redox signaling for an old antioxidant. Frontiers in Pharmacology, 5, 1–12. 10.3389/fphar.2014.00196 25206336 PMC4144092

[phy270016-bib-0005] Chiao, Y. A. , Chakraborty, A. D. , Light, C. M. , Tian, R. , Sadoshima, J. , Shi, X. , Gu, H. , & Lee, C. F. (2021). NAD^+^ redox imbalance in the heart exacerbates diabetic cardiomyopathy. Circulation. Heart Failure, 14, e008170. 10.1161/CIRCHEARTFAILURE.120.008170 34374300 PMC8373812

[phy270016-bib-0006] De Castro, F. (1926). Sur la structure et l'innervation de la glande intercarotidienne (glomuscaroticum) de l'homme et des mammifères, et sur un nouveausystèmed'innervationautonome du nerfglosopharyngien. Travail Laboratoire Recherche Biologique, 24, 365–432.

[phy270016-bib-0007] Diaz Vivancos, P. , Wolff, T. , Markovic, J. , Pallardo, F. V. , & Foyer, C. H. (2010). A nuclear glutathione cycle within the cell cycle. The Biochemical Journal, 431, 169–178. 10.1042/BJ20100409 20874710

[phy270016-bib-0008] Eyzaguirre, C. , & Zapata, P. (1984). Perspectives in carotid body research. Journal of Applied Physiology: Respiratory, Environmental and Exercise Physiology, 57, 931–957. 10.1152/jappl.1984.57.4.931 6150019

[phy270016-bib-0009] Forman, H. J. , Zhang, H. , & Rinna, A. (2009). Glutathione: Overview of its protective roles, measurement, and biosynthesis. Molecular Aspects of Medicine, 30, 1–12. 10.1016/j.mam.2008.08.006 18796312 PMC2696075

[phy270016-bib-0010] Gao, L. , Ortega‐Saenz, P. , Garcia‐Fernandez, M. , Gonzalez‐Rodriguez, P. , Caballero‐Eraso, C. , & Lopez‐Barneo, J. (2014). Glucose sensing by carotid body glomus cells: Potential implications in disease. Frontiers in Physiology, 398, 1–9. 10.3389/fphys.2014.00398 PMC4197775 25360117

[phy270016-bib-0011] Garcia‐Ruiz, C. , Colell, A. , Morales, A. , Kaplowitz, N. , & Fernandez‐Checa, J. C. (1995). Role of oxidative stress generated from the mitochondrial electron transport chain and mitochondrial glutathione status in loss of mitochondrial function and activation of transcription factor nuclear factor kappa‐b: Studies with isolated mitochondria and rat hepatocytes. Molecular Pharmacology, 48, 825–834. 7476912

[phy270016-bib-0012] Ghafourifar, P. , & Saavedra‐Molina, A. (2005). Functions of mitochondrial nitric oxide synthase. In S. Lamas & E. Cadenas (Eds.), Nitric oxide, cell signaling, and gene expression. 1st (pp. 77–98). Taylor & Francis.

[phy270016-bib-0013] Giustarini, D. , Tsikas, D. , Colombo, G. , Milzani, A. , Dalle‐Donne, I. , Fanti, P. , & Rossi, R. (2016). Pitfalls in the analysis of the physiological antioxidant glutathione (GSH) and its disulfide (GSSG) in biological samples: An elephant in the room. Journal of Chromatography. B: Analytical Technologies in the Biomedical and Life Sciences, 1019, 21–28. 10.1016/j.jchromb.2016.02.015 26905452 PMC4829456

[phy270016-bib-0014] Gores, G. J. , Flarsheim, C. E. , Dawson, T. L. , Nieminen, A. L. , Herman, B. , & Lemasters, J. J. (1989). Swelling, reductive stress, and cell death during chemical hypoxia in hepatocytes. The American Journal of Physiology, 257, C347–C354. 10.1152/ajpcell.1989.257.2.C347 2764095

[phy270016-bib-0015] Guarner, V. , & Alvarez‐Buylla, R. (1991). Changes in brain glucose retention produced by the stimulation of an insulin‐sensitive reflexogenic zone in rats. Journal of the Autonomic Nervous System, 34, 89–94. 10.1016/0165-1838(91)90011-q 1940020

[phy270016-bib-0016] Hernandez‐Leal, A. , Tejeda‐Chavez, H. R. , Montero, S. , Lemus, M. , Castro, E. , Ramirez‐Flores, M. , & Roces de Alvarez‐Buylla, E. (2018). Expression of neuronal NO synthase and the hyperglycemic reflex to anoxic stimulation of the carotid body in normoglycemic and hyperglycemic rats. Neurophysiology, 50, 93–98. 10.1007/s11062-018-9722-6

[phy270016-bib-0017] Joyner, M. J. , Limberg, J. K. , Wehrwein, E. A. , & Johnson, B. D. (2018). Role of the carotid body chemoreceptors in glucose homeostasis and thermoregulation in humans. The Journal of Physiology, 596, 3079–3085. 10.1113/jp274354 29377157 PMC6068216

[phy270016-bib-0018] Koyama, Y. , Coker, R. H. , Stone, E. E. , Lacy, D. B. , Jabbour, K. , Williams, P. E. , & Wasserman, D. H. (2000). Evidence that carotid bodies play an important role in glucoregulation in vivo. Diabetes, 49, 1434–1442. 10.2337/diabetes.49.9.1434 10969826

[phy270016-bib-0019] Kumar, P. , & Prabhakar, N. R. (2012). Peripheral chemoreceptors: Function and plasticity of the carotid body. Comprehensive Physiology, 2, 141–219. 10.1002/cphy.c100069 23728973 PMC3919066

[phy270016-bib-0020] Likidlilid, A. , Patchanans, N. , Poldee, S. , & Peerapatdit, T. (2007). Glutathione and glutathione peroxidase in type 1 diabetic patients. Journal of the Medical Association of Thailand, 90, 1759–1767. 17957916

[phy270016-bib-0021] Lopez‐Barneo, J. , Ortega‐Saenz, P. , Pardal, R. , Pascual, A. , Piruat, J. I. , Duran, R. , & Gomez‐Diaz, R. (2009). Oxygen sensing in the carotid body. Annals of the New York Academy of Sciences, 1177, 119–131. 10.1111/j.1749-6632.2009.05033.x 19845614

[phy270016-bib-0022] Lowry, O. H. , Rosebrough, N. J. , Farr, A. L. , & Randall, R. J. (1951). Protein measurement with the folin phenol reagent. The Journal of Biological Chemistry, 193, 265–275. 14907713

[phy270016-bib-0023] Luc, K. , Schramm‐Luc, A. , Guzik, T. J. , & Mikolajczyk, T. P. (2019). Oxidative stress and inflammatory markers in prediabetes and diabetes. Journal of Physiology and Pharmacology, 70, 809–824. 10.26402/jpp.2019.6.01 32084643

[phy270016-bib-0024] Lushchak, V. I. (2012). Glutathione homeostasis and functions: Potential targets for medical interventions. Journal of Amino Acids, 2012, 736–837. 10.1155/2012/736837 PMC3303626 22500213

[phy270016-bib-0025] Ma, W. X. , Li, C. Y. , Tao, R. , Wang, X. P. , & Yan, L. J. (2020). Reductive stress‐induced mitochondrial dysfunction and cardiomyopathy. Oxidative Medicine and Cellular Longevity, 2020, 5136957. 10.1155/2020/5136957 32566086 PMC7277050

[phy270016-bib-0026] Mari, M. , Morales, A. , Colell, A. , Garcia‐Ruiz, C. , & Fernandez‐Checa, J. (2009). Mitochondrial glutathione, a key survival antioxidant. Antioxidants & Redox Signaling, 11, 2685–2700. 10.1089/ARS.2009.2695 19558212 PMC2821140

[phy270016-bib-0027] McDonald, D. M. , & Blewett, R. W. (1981). Location and size of carotid body‐like organs (paraganglia) revealed in rats by the permeability of blood vessels to Evans blue dye. Journal of Neurocytology, 10, 607–643. 7310468 10.1007/BF01262593

[phy270016-bib-0028] Moree, S. S. , Kavishankar, G. B. , & Rajesha, J. (2013). Antidiabetic effect of secoisolariciresinol diglucoside in streptozotocin‐induced diabetic rats. Phytomedicine, 20, 237–245. 10.1016/j.phymed.2012.11.011 23271000

[phy270016-bib-0029] Ortiz‐Avila, O. , Esquivel‐Martinez, M. , Olmos‐Orizaba, B. E. , Saavedra‐Molina, A. , Rodriguez‐Orozco, A. R. , & Cortes‐Rojo, C. (2015). Avocado oil improves mitochondrial function and decreases oxidative stress in brain of diabetic rats. Journal Diabetes Research, 2015, 1–9. 10.1155/2015/485759 PMC4477098 26180820

[phy270016-bib-0030] Pardal, R. , & Lopez‐Barneo, J. (2002). Low glucose–sensing cells in the carotid body. Nature Neuroscience, 5, 197–198. 10.1038/nn812 11850631

[phy270016-bib-0031] Perez‐Gallardo, R. V. , Noriega‐Cisneros, R. , Esquivel‐Gutierrez, E. , Calderon‐Cortes, E. , Cortes‐Rojo, C. , Manzo‐Avalos, S. , Campos‐Garcia, J. , Salgado‐Garciglia, R. , Montoya‐Perez, R. , Boldogh, I. , & Saavedra‐Molina, A. (2014). Effects of diabetes on oxidative and nitrosative stress in kidney mitochondria from aged rats. Journal of Bioenergetics and Biomembranes, 46, 511–518. 10.1007/s10863-014-9594-4 25425473

[phy270016-bib-0032] Peris, E. , Micallef, P. , Paul, A. , Palsdottir, V. , Enejder, A. , Bauza‐Thorbrugge, M. , Olofson, C. S. , & Asterholm, I. W. (2019). Antioxidant treatment induces reductive stress associated with mitochondrial dysfunction in adipocytes. The Journal of Biological Chemistry, 294, 2340–2352. 10.1074/jbc.RA118.004253 30559295 PMC6378980

[phy270016-bib-0033] Poret, J. M. , Gaudet, D. A. , Braymer, H. D. , & Primeaux, S. D. (2021). Sex differences in markers of metabolic syndrome and adipose tissue inflammation in obesity‐prone, Osborne‐Mendel and obesity‐resistant, S5B/Pl rats. Life Sciences, 273, 119–290. 10.1016/j.lfs.2021.119290 PMC9594853 33662430

[phy270016-bib-0034] Powers, A. C. , Niswender, K. D. , & Evans‐Molina, C. (2022). Diabetes mellitus: Diagnosis, classification, and pathophysiology. In J. Loscalzo , A. Fauci , D. Kasper , et al. (Eds.), Harrison's principles of internal medicine. 21st (pp. 3094–3102). McGraw‐Hill.

[phy270016-bib-0035] Saavedra‐Molina, A. , & Devlin, T. M. (1997). Effect of extra‐ and intra‐mitochondrial calcium on citrulline synthesis. Amino Acids, 12, 293–298.

[phy270016-bib-0036] Sanchez‐Duarte, S. , Marquez‐Gamino, S. , Montoya‐Perez, R. , Villicana‐Gomez, E. A. , Vera‐Delgado, K. S. , Caudillo‐Cisneros, C. , Sotelo‐Barroso, F. , Melchor‐Moreno, M. T. , & Sanchez‐Duarte, E. (2021). Nicorandil decreases oxidative stress in slow‐ and fast‐twitch muscle fibers of diabetic rats by improving the glutathione system functioning. Journal of Diabetes Investigation, 12, 1152–1161. 10.1111/jdi.13513 33503290 PMC8264387

[phy270016-bib-0037] Sanz‐Alfayate, G. , Obeso, A. , Agapito, M. T. , & Gonzalez, C. (2001). Reduced to oxidized glutathione ratios and oxygen sensing in calf and rabbit carotid body chemoreceptor cells. The Journal of Physiology, 537, 209–220. 10.1111/j.1469-7793.2001.0209k.x 11711574 PMC2278940

[phy270016-bib-0038] Sims, N. R. (1990). Rapid isolation of metabolically active mitochondria from rat brain and sub‐regions using percoll density gradient centrifugation. Journal of Neurochemistry, 55, 698–707. 10.1111/j.1471-4159.1990.tb04189.x 2164576

[phy270016-bib-0039] Singh, F. , Charles, A. L. , Schlagowski, A. I. , Bouitbir, J. , Bonifacio, A. , Piquard, F. , Krahenbuhl, S. , Geny, B. , & Zoll, J. (2015). Reductive stress impairs myoblasts mitochondrial function and triggers mitochondrial hormesis. Biochimica et Biophysica Acta, 1853, 1574–1585. 10.1016/j.bbamcr.2015.03.006 25769432

[phy270016-bib-0040] Skyler, J. S. , Bakris, G. L. , Bonifacio, E. , Darsow, T. , Eckel, R. H. , Groop, L. , Groop, P. H. , Handelsman, Y. , Insel, R. A. , Mathieu, C. , McElvaine, A. T. , Palmer, J. P. , Pugliese, A. , Schatz, D. A. , Sosenko, J. M. , Wilding, J. P. H. , & Ratner, R. E. (2017). Differentiation of diabetes by pathophysiology, natural history, and prognosis. Diabetes, 66, 241–255. 10.2337/db16-0806 27980006 PMC5384660

[phy270016-bib-0041] Szkudelski, T. (2001). The mechanism of alloxan and streptozotocin action in B cells of the rat pancreas. Physiological Research, 50, 537–546. 11829314

[phy270016-bib-0042] Tejeda‐Chavez, H. R. , Montero, S. A. , Lemus, M. , Leal, C. A. , Portilla‐de Buen, E. , Hernandez, A. G. , & Roces de Alvarez‐Buylla, E. (2010). Concomitant effects of nitric oxide and carotid chemoreceptor stimulation on brain glucose in normoglycemic and hyperglycemic rats. Archives of Medical Research, 41, 487–496. 10.1016/j.arcmed.2010.09.008 21167387

[phy270016-bib-0043] Wall, S. B. , Smith, M. R. , Ricart, K. , Zhou, F. , Vayalil, P. K. , Oh, J. Y. , & Landar, A. (2014). Detection of electrophile‐sensitive proteins. Biochimica et Biophysica Acta, 1840, 913–922. 10.1016/j.bbagen.2013.09.003 24021887 PMC3858512

[phy270016-bib-0044] Waynfort, H. B. , & Flecknell, P. A. (Eds.). (1995). Experimental and surgical technique in the rat. Academic Press.

[phy270016-bib-0045] Wendel, A. (1987). Measurement of in vivo lipid peroxidation and toxicological significance. Free Radical Biology & Medicine, 3, 355–358. 10.1016/s0891-5849(87)80047-3 3319801

[phy270016-bib-0046] Wu, J. , Jin, Z. , & Yan, L. J. (2017). Redox imbalance and mitochondrial abnormalities in the diabetic lung. Redox Biology, 11, 51–59. 10.1016/j.redox.2016.11.003 27888691 PMC5124358

[phy270016-bib-0047] Xiao, W. , & Loscalzo, J. (2020). Metabolic responses to reductive stress. Antioxidants & Redox Signaling, 32, 1330–1347. 10.1089/ars.2019.7803 31218894 PMC7247050

[phy270016-bib-0048] Yu, Q. , Lee, C. F. , Lee, C. F. , Wang, W. , Karamanlidis, G. , Kuroda, J. , Matsushima, S. , Sadoshima, J. , & Tian, R. (2014). Elimination of NADPH oxidase activity promotes reductive stress and sensitizes the heart to ischemic injury. Journal of the American Heart Association, 3, 1–16. 10.1161/JAHA.113.000555 PMC3959718 24470522

[phy270016-bib-0049] Zhang, H. , Limphong, P. , Pieper, J. , Liu, Q. , Rodesch, C. K. , Christians, E. , & Benjamin, I. J. (2012). Glutathione‐dependent reductive stress triggers mitochondrial oxidation and cytotoxicity. The FASEB Journal, 26, 1442–1451. 10.1096/fj.11-199869 22202674 PMC3316899

[phy270016-bib-0050] Zhang, P. , Li, S. , Guo, Z. , & Lu, S. (2019). Nitric oxide regulates glutathione synthesis and cold tolerance in forage legumes. Environmental and Experimental Botany, 167, 1–8. 10.1016/j.envexpbot.2019.103851

